# Food packaging cues influence taste perception and increase effort provision for a recommended snack product in children

**DOI:** 10.3389/fpsyg.2015.00882

**Published:** 2015-07-02

**Authors:** Laura Enax, Bernd Weber, Maren Ahlers, Ulrike Kaiser, Katharina Diethelm, Dominik Holtkamp, Ulya Faupel, Hartmut H. Holzmüller, Mathilde Kersting

**Affiliations:** ^1^Department of Epileptology, University Hospital BonnBonn, Germany; ^2^Department of NeuroCognition/Imaging, Life and Brain CenterBonn, Germany; ^3^Center for Economics and Neuroscience, University of BonnBonn, Germany; ^4^Research Institute of Child Nutrition, University of BonnDortmund, Germany; ^5^Department of Marketing, University of DortmundDortmund, Germany

**Keywords:** child marketing, reinforcing value, placebo effect, healthy food, nutritional requirements

## Abstract

Food marketing research shows that child-directed marketing cues have pronounced effects on food preferences and consumption, but are most often placed on products with low nutritional quality. Effects of child-directed marketing strategies for healthy food products remain to be studied in more detail. Previous research suggests that effort provision explains additional variance in food choice. This study investigated the effects of packaging cues on explicit preferences and effort provision for healthy food items in elementary school children. Each of 179 children rated three, objectively identical, recommended yogurt-cereal-fruit snacks presented with different packaging cues. Packaging cues included a plain label, a label focusing on health aspects of the product, and a label that additionally included unknown cartoon characters. The children were asked to state the subjective taste-pleasantness of the respective food items. We also used a novel approach to measure effort provision for food items in children, namely handgrip strength. Results show that packaging cues significantly induce a taste-placebo effect in 88% of the children, i.e., differences in taste ratings for objectively identical products. Taste ratings were highest for the child-directed product that included cartoon characters. Also, applied effort to receive the child-directed product was significantly higher. Our results confirm the positive effect of child-directed marketing strategies also for healthy snack food products. Using handgrip strength as a measure to determine the amount of effort children are willing to provide for a product may explain additional variance in food choice and might prove to be a promising additional research tool for field studies and the assessment of public policy interventions.

## Introduction

Food preferences and dietary habits develop at an early age and are one of many determinants for the development of obesity later in life (Birch and Fisher, 1998; Benton, 2004; Harris, 2008; Beauchamp and Mennella, 2009). Children's nutrition and nutritional status can affect adult health (Gelperowic and Beharrell, 1994; Dietz, 1998; Biro and Wien, 2010) and has therefore important long-term implications for individual development as well as national healthcare systems. However, in many industrialized countries, there is a major gap between dietary recommendations and actual food choices in the population, with obesity already highly prevalent in children (e.g., James et al., 2001; Janssen et al., 2005; Rosario et al., 2010; Flegal et al., 2012). This imbalance between dietary recommendations and actual food-intake can at least partially be explained by an obesogenic environment that promotes high amounts of energy-dense food products to children (Lobstein and Dibb, 2005; Halford et al., 2008). Research points to a link between the food industry's marketing strategies targeted at children and increased prevalence of childhood obesity (Halford et al., 2007; Linn and Novosat, 2008; Bruce et al., 2014). No et al. (2014) analyzed marketing strategies in magazines targeted at children and adolescents and found that food marketing clearly skews toward promoting unhealthy food products (No et al., 2014). Children are an attractive target group for the food industry with a rising number of food products especially customized and advertised to children (e.g., Hastings et al., 2003; Lobstein and Dibb, 2005; Linn and Novosat, 2008; Harris et al., 2009, 2010). This is of serious concern because most of the advertised food products do not meet the dietary guidelines that recommend only sparse amounts of energy-dense, high-fat, and high-sugar products and high amounts of fruit and vegetables (Kersting et al., 2005; No et al., 2014).

A recent systematic review concluded that companies use characters on products to build an emotional relationship between children and products (Kraak and Story, 2015). Children are especially susceptible to marketing effects as they are less skeptical about its persuasive intent (Roberto et al., 2010). Cross-media advertising may even increase the severity of these effects (Kelly et al., 2015). It has been repeatedly shown that marketing actions directed at children are successful in affecting recognition, popularity and request of both healthy and unhealthy products (De Droog et al., 2011; Cairns et al., 2013; Reisch et al., 2013; Jenkin et al., 2014). Important goals to reduce obesity are to limit the extent and the persuasive power of children's exposure to marketing for unhealthy food items (Kraak and Story, 2015). A conceptual model presented by the IOM Food Marketing Committee (National Research Council, 2006) proposed a causal mechanism by which marketing affects diet and health outcomes. Exogenous marketing variables, such as product packaging and portion size, affect diet via several mediators. Mediators are for example the change of purchase request, preferences and beliefs. Diet in turn impacts long-term health outcomes, such as obesity or metabolic syndrome. The model also takes into account moderating factors, such as gender, socioeconomic status and age and provides a framework for empirical research (National Research Council, 2006). Here, we analyze the causal connection between marketing and preferences, that is, liking and motivation to work for an item, which are mediating factors in the proposed conceptual model.

Inherently, food high in sugar and fat is often preferred over less energy-dense food (Drewnowski and Greenwood, 1983; Stice et al., 2013a). Children may not be intrinsically attracted to healthy food products, especially those that lack “child-appeal” (Gelperowic and Beharrell, 1994). Given the potential of fun characters to influence children's taste preference and request for food products, target-specific marketing cues may be a promising tool to also promote healthy food products (Roberto et al., 2010, De Droog et al., 2011; Wansink et al., 2012a). Several institutions even recommend that cartoon characters may only be used to promote healthy food products that are consistent with science-based nutritional standards (National Research Council, 2006; White House Task Force on Childhood Obesity, 2010; Kraak and Story, 2015). De Droog and colleagues found that brand characters on healthy food products can increase package liking and purchase requests of the products up to a level similar to candy, but they did not elicit taste ratings (De Droog et al., 2011). Attractive names for raw vegetables and vegetable dishes could persistently increase healthy food consumption in children (Wansink et al., 2012b). Possibly, the effect of child-directed marketing actions on nutritionally recommended products may be due to an increase in attractiveness of the product and therefore a more favorable attitude toward the respective products (Roberto et al., 2010).

Marketing actions that modify peripheral components of products, such as packaging or labels, can induce so-called “placebo effects,” that is, altering experienced pleasantness and efficacy of an otherwise identical product (Shiv et al., 2005; Plassmann and Weber, in press). For example, in adults, a higher price was shown to increase taste pleasantness of an identical product (Plassmann et al., 2008), and also influence behavioral performance measures (Shiv et al., 2005). Roberto and colleagues demonstrated that children preferred the taste of foods that had popular cartoon characters on the packaging, compared to the same foods with bland packaging (Roberto et al., 2010). Similarly, we expect increased experienced taste pleasantness ratings for yogurts presented in a package that includes an unknown cartoon character.

Previous studies suggest that liking measures do not fully explain food choices (Epstein et al., 2003; Mela, 2006; Temple, 2014). In contrast to subjective liking scales, the motivation to work for an item, that is, its reinforcing value, can be objectively measured by determining how much work an individual engages in to receive an item (Temple, 2014). The motivation to work for an item can be assessed for example by observing lever presses or other motor responses (Bower and Kaufman, 1963; Saelens and Epstein, 1996; Epstein et al., 2007; Temple, 2014). In a very influential model of reward processing, Berridge distinguished between liking and wanting components (Berridge, 1996; Berridge and Robinson, 2003; Berridge and Kringelbach, 2008). While liking relates to the hedonic impact of a reward, that is, the pleasure it elicits, wanting describes the motivational component of a reward, that is, the desire to obtain it (Berridge, 1996; Finlayson and Dalton, 2012). Although reinforcing food products are often also liked, it is possible to empirically dissociate between liking and wanting, as certain factors alter only liking, but not wanting of food products, or vice versa (Temple, 2014). For example, previous studies showed that obesity is not reliably associated with heightened explicit liking of products, but may be associated with increased motivation for food consumption (Mela, 2006). Moreover, food deprivation selectively influences food wanting but not liking (Epstein et al., 2003). Justification for the distinction between liking and wanting components also stems from neurobiological studies, as separate neural pathways exist to mediate these processes (Finlayson and Dalton, 2012). Liking seems to relate to the opioid circuitry, while wanting seems to relate to the mesolimbic dopamine system (Berridge and Robinson, 2003). Separating the components of liking and wanting may be crucial to further elucidate factors that motivate people to eat (Epstein et al., 2007). Determining how to increase motivation for less liked foods, for instance, may improve food choice (Temple, 2014).

Regarding the results that liking and wanting components may be separate measures that explain choices, more work is needed to understand the links between liking, wanting and food choice, and how to reliably measure them. Thus, far, research on effort provision measurements, which were shown to provide information above and beyond self-report, have been confined to adults. In this study, we used a method not routinely used up to now to quantify the motivation to work for the respective food products, i.e., handgrip strength. Grip strength measures are routinely used in rehabilitation and clinical settings, and have been shown to be reliable and simple to perform (Innes, 1999). For instance, maximum strength is used to assess hand function, strength, or nutritional status (Pieterse et al., 2002; Molenaar et al., 2010). It is also possible to assess relative grip strength of individuals, by dividing expended strength for a specific item by individual maximum handgrip strength. In a study investigating the effect of subliminal primes on motivation and effort provision, Aarts and colleagues showed that reward-cues influenced the speed of motor reactions and the effort subjects were spending, even though the subjects were neither consciously aware of them nor explicitly instructed to apply different levels of effort (Aarts et al., 2008). Using handgrip dynamometers is also feasible for children (Molenaar et al., 2010). Grip strength measures provide an objective measure for the amount of work an individual is willing to expend in order to receive an item. Fun characters may not only induce a taste-placebo effect, but also increase the motivation to work for an identically composed snack product. The aim of this measure was to determine the effort that children were willing to provide for the respective products. Additionally, it is of interest whether liking and effort measures can independently explain variance in food choice.

The objective of this study was to investigate the effects of different labels on a nutritionally optimized snack-meal on different measures of preferences in children. Explicit liking, that is, taste ratings and preference, was measured using questionnaires. We used a novel approach to measure the motivation to work for food products in children, namely a handgrip dynamometer. The strength measure provides an objective measure for effort provision. We hypothesized that the integration of unknown fun characters on the packaging of the healthy snacks leads to an increase in explicitly stated taste pleasantness. As only the label was manipulated while the product's composition was identical, the observed effects on taste can be interpreted as placebo effects only. Further, we tested the handgrip dynamometer as a novel tool in this domain and expected increased effort provision for the fun labeled product. Also, we investigated whether both measures separately explained variance in choice behavior.

## Methods

### Study sample

This study was conducted in samples of school children in four primary schools in Dortmund, Germany, in 2012 and 2014, with children of the 3rd and 4th grade (aged 8–10 years). We chose this timing (i.e., a year gap between the measures) for practical reasons to measure two different cohorts in the same schools. Inclusion criteria were a parental signed consent and participation in anthropometric measurements and all product tests. The final sample comprised 179 children, 82 in 2012 and 97 in 2014. The study was approved by the ethical committee of the University of Bonn.

### General measures

Body height and weight were measured to the nearest 0.1 cm and 0.1 kg, respectively, using portable stadiometers and digital scales (Seca 225 and 704; Seca, Hamburg, Germany) by trained staff, with participants in light clothing and without shoes to calculate the individual body mass index (BMI, kilogram/meter^2^). Handgrip strength was used to elicit the motivation to work for the respective items. A few days prior to product testing, maximum grip strength was measured for the non-dominant hand with an adjustable handgrip dynamometer (Jamar 5030 J1, Jackson, Michigan USA) that was adjusted according to each child's hand size (Ortega et al., 2008). Children were asked to grip as strongly as possible (stated in kilogram, kg). This procedure was repeated three times with sufficient breaks to ease the hand. The maximum of the three contractions was used as individual maximum handgrip strength for subsequent analyses to control for individual differences in strength.

### Yoghurt tasting and effort provision task

Three yogurts in transparent plastic containers with different labels were presented to the children. Each container was filled with the same yogurt (120 g) consisting of semi-skimmed yogurt (40%), puréed red fruits (mostly cherries and strawberries, 35%) and whole-grain cereals in the form of small stars (25%) (WILD Dairy Ingredients GmbH, Heidelberg, Germany). Composition was in accordance with the snack meal within the Optimized Mixed Diet (*optiMIX*), a meal-based preventive dietary concept developed by the Research Institute of Child Nutrition (Kersting et al., 2005).

**Figure 1:**
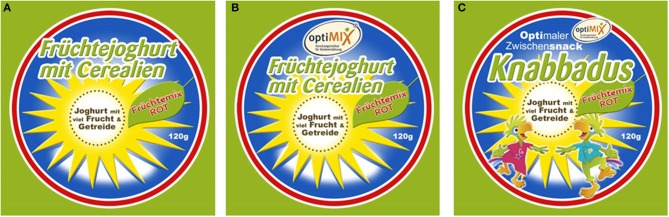
**Illustration of the labels on top of the food packaging. (A)** Plain label; **(B)** Health Label, **(C)** Fun Label. Translation of the labels: Header: fruit yogurt with cereals. Text within the yellow star: yogurt with a lot of fruit and cereals. Text on the green leaf: Fruit mix red. Additional information on the second and third label on top: OptiMix—Research institute for child nutrition. Additional information on the third label next to the OptiMix emblem: Optimal snack.

The labels for the *optiMIX*-snacks were designed as follows (see also Figure 1):

- “Plain label”: the label depicted the name of the snack (yogurt-fruit-mix) on a yellow star.
- “Health label”: the label depicted the name of the snack on a yellow star and additionally the *optiMIX* label, marking the snack as corresponding to the guidelines of the Optimized Mixed Diet.
- “Fun label”: in addition to the features of the health label, this label included the notion “optimal snack” and two blue cockatoos. Further, the product name was changed to a more child-directed name (“Knabbadus”).

We chose to use the combined label (for III) to create a packaging that would be directed at children as well as at parents. Interaction effects between the fun character and the health information are addressed in the discussion. Children were asked whether they liked yogurt in general and were given the option not to participate. Only children who took part in both the taste and effort provision experiment were included in the analyses. Children were asked to rate their subjective hunger level on a 5-point scale ranging from “very full” to “very hungry.” The standardized product testing protocol proceeded as follows: Groups of several children were seated in a separated schoolroom, each child at a separate table, and were assisted individually by one trained member of staff while conducting the study protocol. The products I–III were presented in a randomized order to each participant. The children were asked to take the dynamometer, look at the different snacks and press the dynamometer as much as they would like to have each product. The obtained values were employed to calculate percentages of maximum grip strength for the following analyses. The children were asked to open each snack container, to sample it, and rate the taste on a 7-point scale (“extremely bad,” “very bad,” “bad,” “indifferent,” “good,” very good” and “extremely good”). They could not see the evaluation of the other children in the group. In both cohorts, children received the same task instruction; they were instructed to press as much as they wanted to have the product. In 2012, children received the product for which they pressed most. In 2014, though, children could choose whichever product they preferred after the sensory taste test. We used the 2014 choices to investigate the differential effects of taste pleasantness and effort provision on choice. It is important to note, however, that this choice measure is not fully independent of the previous tests.

### Statistical analysis

Data were analyzed using R Studio (R Studio: Integrated development environment for R, Version 0.97.551, Boston, MA, USA) and SPSS (IBM SPSS Statistics for Windows, Version 21.0. Armonk, NY, USA). Normality tests were calculated. Since all our data violated the assumption of normality, Fridman tests for analyses of variance, and Wilcoxon-tests for pairwise comparisons were used. Multiple tests were Bonferroni-corrected. We correlated liking and handgrip measures for each label (Kendall's Tau rank correlation). As handgrip measures were highly variable and lacked a common anchor (such as a boundary or a mean), we also correlated taste and effort placebo effects.

As hunger status was shown to be an important component that determines food choice (Rogers and Hardman, 2015), we also performed linear mixed-model analyses, with liking (model 1) or effort provision (model 2) as dependent variable, label and hunger level as predictors, and subject as random effect. We tested whether hunger moderated or mediated the effect of label on the measures by adding an interaction term for hunger and label (for the moderator analysis), and by using the R “mediation” package (for the mediation analysis).

In 2014, we let the children choose a yogurt independent of their effort. We used a logistic regression analysis with both placebo effects as predictors and whether they chose the fun labeled product as a binary dependent variable. Using raw effort measure values rather than the difference between two effort values (i.e., the effort placebo effect measured as the difference between fun labeled and plain product) is rather difficult, as there is no clear boundary for the effort value and the intra- and inter-individual variance is very high. We tested whether adding the effort provision and liking placebo values reduced deviance in the model to explain choice behavior.

## Results

Characteristics of the sample are presented in Table 1. Children were between 8 and 10 years old. There were no significant gender differences in age and BMI. Absolute maximal handgrip strength differed significantly between boys and girls. However, further analyses were conducted using relative handgrip strength, that is, hand grip strength for the respective labels as percentage of maximum handgrip strength. Hence, differences between boys and girls were considered a negligible factor and data for boys and girls were pooled for further analyses.

**Table 1:** **Characteristics of the participants (*****N*** **= 179)**.

**Variables**	**Boys (*n* = 87, 48.6%)**	**Girls (*n* = 92, 51.4%)**	***p* for difference^a^**
Age (years)	9.0 (1)	9.0 (1)	0.09
Body mass index (kg/m^2^)	16.2 (2.9)	16.4 (3.2)	0.62
Dominant hand right (left)	93.6%	95.5%	0.52
Maximal hand grip strength (kg)	14.0 (4.7)	13.0 (5)	0.01

*Values are presented as medians (interquartile range) or percentages in case of categorical variables*.

a *Significant differences tested using Wilcoxon-Tests for non-normally distributed interval and ordinal data and Chi-Square-Test for categorical variables*.

**Figure 2:**
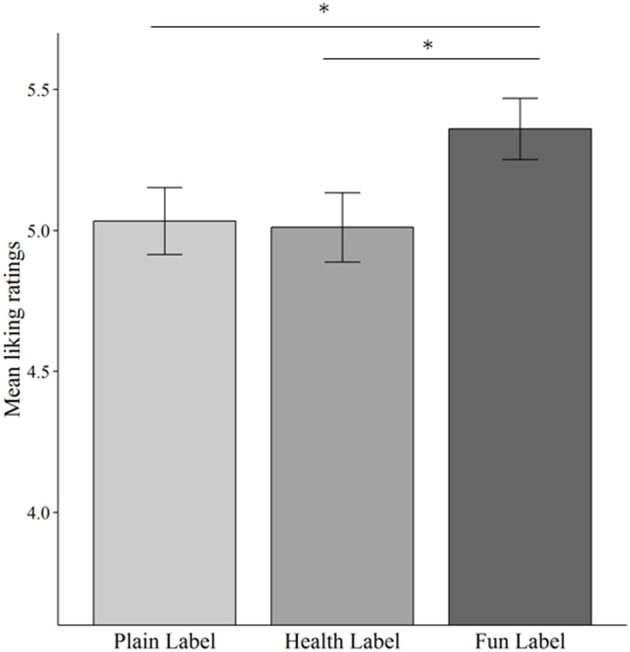
**Mean liking ratings of the snack presented with different packaging cues**. Error bars indicate standard error of the mean. ^*^p < 0.05.

There was a statistically significant difference in stated taste pleasantness, depending on the label [χ^2^(2) = 7.129, *p* = 0.028]. 88% of children stated different taste liking ratings for the objectively equivalently composed products. Taste pleasantness was significantly higher for the fun label compared to the plain and health label; see Figure 2 and Table 2. Children were asked which yogurt they preferred. 56.3% of children preferred the fun label snack (plain label: 21.8%; health label: 21.8%).

**Table 2:** **Explicit liking of and effort provision for the presented products (*****N*** **= 179)**.

		**Explicit liking (taste ratings)**	**Effort provision (% of maximum handgrip strength)**
Friedman test	Overall	**χ^2^(2) = 7.129, *p* = 0.028**	**χ^2^(2) = 15.678, *p* < 0.001**
Median (interquartile range)	Plain label	5 (2)	66.7 (42.8)
	Health label	5 (2)	66.7 (38.3)
	Fun label	6 (2.3)	75.0 (37.9)
Mean (standard deviation)	Plain label	5.03 (1.58)	64.61 (34.42)
	Health label	5.01 (1.64)	63.28 (29.03)
	Fun label	5.36 (1.45)	72.41 (30.15)
Wilcoxon test	Health label—Plain label	Z = –0.716, *p* = 0.716	Z = –0.744, *p* = 0.457
	Fun label—Plain label	**Z** = –**2.759**, ***p*** = **0.005**	**Z** = –**3.220**, ***p*** = **0.001**
	Fun label—Health label	**Z** = –**2.972**, ***p*** = **0.003**	**Z** = –**3.798**, ***p*** < **0.001**

*Due to non-normality of data, non-parametric tests were applied. Post-hoc analysis with Wilcoxon signed-rank tests was conducted with a Bonferroni correction applied. The resulting significance level was set at p < 0.017. Bold print indicates that the respective significance level was reached*.

**Figure 3:**
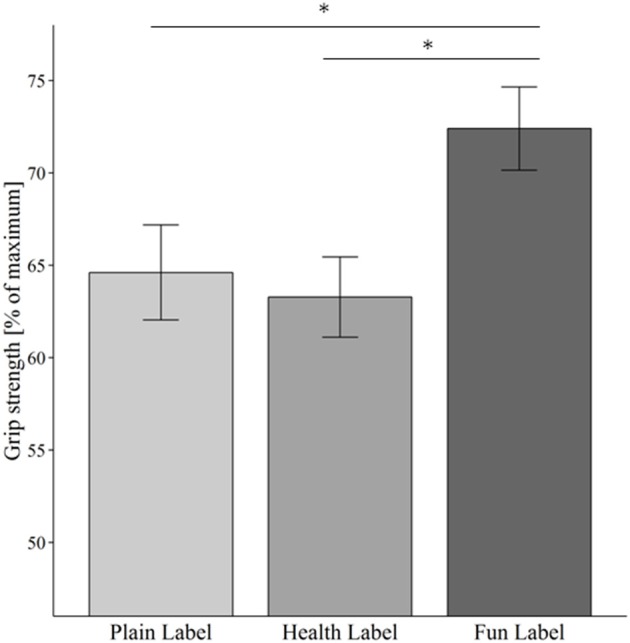
**Mean hand grip strength: Hand grip strength relative to the individual maximum strength applied by the children for the products with different packaging cues as an indicator for motivation to obtain the respective food item**. Error bars indicate standard error of the mean. ^*^p < 0.05.

When looking at the effort provision measure, children showed a statistically significant difference in percentage of exerted maximum handgrip strength depending on the packaging label [χ^2^(2) = 15.678, *p* < 0.001]. Median percentage [interquartile range] of maximum handgrip strength was 66.7 [42.8] for the plain label, 66.7 [38.3] for the health label, and 75.0 [37.9] for the fun label. There was a significant difference in handgrip strength between the fun label and the plain label and between the fun label and the health label, see Figure 3 and Table 2. Results were very similar when children that stated that they “did not really” (*n* = 5) or “did not like” (*n* = 1) yogurt were excluded.

Only in the second cohort (in 2014, *n* = 89) we also let the children choose one of the yogurts to take home at the end of the experiment. To test whether liking and effort separately explain variance in food choice, we used a logistic regression analysis, with fun labeled product chosen (yes/no) as the dependent variable, and effort and liking placebo effects as predictors. We then tested whether adding effort provision and liking reduced deviance in the model to explain choice behavior. Indeed, effort provision placebo effects as well as taste placebo effects reduced deviance in the model (Effort: residual deviance = 110.18, *df* = 87, *p* = 0.01; taste: residual deviance = 101.17, *df* = 86, *p* = 0.003). Further, we checked for multicollinearity, that is, the dependence of predictors. We found that the variance inflation factor was very low (i.e., 1.03), indicating that the predictor variables were not collinear. Residuals were also not correlated (Durbin Watson test statistic = 2, *p* = 0.9).

We also correlated both taste and effort placebo effects with BMI. No measures were significant (all *p*'s > 0.05). Additionally, we correlated liking ratings with the effort provision task across labels. We could not find a significant correlation (Kendall's Tau = 0.04, *p* = 0.2). However, this may be due to the fact that the effort provision task has no common middle value or boundary across participants and the variability in the effort provision task is quite high. We therefore correlated the effort placebo effect and the taste placebo effect. One would expect that children who exhibit a higher effort placebo effect also show a higher taste placebo effect. Indeed, this correlation was significant (Kendall's Tau = 0.173, *p* = 0.002).

Mean hunger level was 2.57 (*SD* = 0.84), which corresponds to “rather full” to “not really hungry/not really full.” In the linear mixed-model analyses, we tested for an effect of hunger level and label on liking ratings (model 1) and effort provision (model 2). In model 1 (liking), hunger levels were significant covariates in the model, with higher liking ratings for higher hunger levels [effect label: *F*_(2, 354)_ = 5.24, *p* = 0.006; effect hunger level: *F*_(1, 176)_ = 13.0, *p* < 0.001]. Hunger levels did not moderate or mediate the effect of label on liking (all *p*'s > 0.5). In model 2 (effort provision), hunger was not a significant covariate in the model, [effect label: *F*_(1, 354)_ = 8.18, *p* < 0.001; effect hunger level: *F*_(1, 176)_ = 1.6, *p* = 0.2]. Again, hunger level did not moderate or mediate the effect of label on effort provision (all *p*'s > 0.5).

## Discussion

This study provides empirical evidence for a causal relationship between marketing cues on food packaging and different measures of children's preferences of an objectively identical healthy snack product. Child-directed packaging cues with unknown cartoon characters enhanced the attractiveness of a healthy snack amongst elementary school children. The fun label snack significantly increased stated taste perception and the motivation to work for the product.

The explicit taste ratings revealed a significant taste placebo effect, that is, increased stated taste-pleasantness of the identical product depending on the packaging. Children reported that the health plus fun labeled product tasted better, compared to the product labeled with a plain or health-only label. This marketing effect is in line with a previous study that showed that children preferred foods that had a popular cartoon character on the packaging (Roberto et al., 2010). Unfortunately, most marketing actions promote tasty energy-dense food products (Roberto et al., 2010). Correlational field data has suggested a relationship between increased sales of vegetables and packaging that included a well-known comic character (Radice, 2007), or an attractive product name (Wansink et al., 2012b). Another study found that child-directed marketing cues increased liking and purchase request for fruits up to a level comparable to highly palatable, energy-dense food products, however, the study did not elicit taste ratings (De Droog et al., 2011). Robinson and colleagues found that vegetables were rated higher by children when presented within a McDonald's meal (Robinson et al., 2007). The utilized McDonalds logo represents a very strong brand, which is very familiar to children. In contrast, we show than even self-created cartoon characters and product names, which were unknown to the children, lead to changes in taste experience. As the label was not formerly introduced to the study group, confounding effects of prior exposure or learned associations can be excluded, except for possibly prior exposure to the OptiMix logo.

In addition to explicit questionnaires, we also applied a handgrip dynamometer to measure the motivation to work for an item. Our results revealed increased expended effort for a fun label character with a child-directed product name compared to a plain or health label. Research on food preferences in children is usually conducted by asking for preference for one product over another, or by eliciting liking ratings (Gorn and Goldberg, 1982; Clarke, 1984; Gorn and Florsheim, 1985; Borzekowski and Robinson, 2001; Liem and Zandstra, 2009; Roberto et al., 2010, Kildegaard et al., 2011; Wansink et al., 2012a). Previous research conducted with adults provides evidence that effort provision explains additional variance of food choice and can therefore provide information above and beyond self-reported measures (Mela, 2001; Epstein et al., 2007; Finlayson et al., 2008; Cameron et al., 2014). For example, studies found a relationship between obesity or food deprivation and increased willingness to work for food rewards, but no relationship between obesity or food deprivation and hedonic ratings of the food rewards (Johnson, 1974; Saelens and Epstein, 1996; Epstein et al., 2003), suggesting independent processes. To our knowledge, wanting of a food product has been previously elicited only by explicit assessments in children (Hill et al., 2009; Liem and Zandstra, 2009, Kildegaard et al., 2011). Explicit wanting, that is, self-reported willingness to work for a candy, compared with non-food rewards, predicted 1-year weight gain in children (Hill et al., 2009). However, explicit assessment of wanting is often not accurate (Finlayson and Dalton, 2012).

Previous research on implicit wanting used for example key pressing tasks with different reinforcement schedules (Epstein et al., 2007). Also, joystick tasks have been used to indirectly capture implicit motivational tendencies (Piqueras-Fiszman et al., 2014). In their task, the authors recorded response latencies while participants had to move a joystick toward or away from themselves according to unrelated instructions, while they also saw food images. They found that food valence interacted with the latency of the movement. In the present study, the handgrip dynamometer was explicitly cued. This measure provides faster measures of expended effort compared to prior tasks. It can be easily applied in group settings and does not require computers or laboratories. The dynamometer is therefore an interesting tool for field studies, such as in a supermarket or school. We show that a simple manipulation of a label can increase effort provision for a recommended snack-food product. However, more research is needed to establish this device as a valid and reliable tool to measure the reinforcing value of food products, also by comparing food products with different nutritional densities and under conditions that influence only liking, but not wanting, and vice versa. An important factor related to food reinforcement is dopaminergic activity (Berridge, 1996; Ng et al., 2011; Stice et al., 2013b). It would therefore be very interesting to further elucidate the neural mechanisms of how labels influence the reinforcing value of food product by combining effort measures with neuroimaging. It is also important to investigate, whether effort provision explains additional and more importantly also significant amounts of variance in food choice. In the second cohort of the present study, children were given the option to choose whichever product they wanted to take home, independent of their previously expended effort. We could show that liking ratings and effort provision separately explained variance in choices. However, this is a rather noisy measure of food choice, as we have to assume dependency between the measures and choice. A more independent measure of food choice is clearly needed and this issue has to be addressed in future studies.

Young children have been shown to be very vulnerable to advertising as they do not understand the persuasive intent behind marketing actions (Roberto et al., 2010). In our study, only 21 out of 179 children stated identical taste liking ratings for all three, objectively identically tasting, products. We also performed correlational analysis with BMI. However, neither taste nor effort provision effects were significant. We suspect that this may be due to the homogeneity of BMIs within the sample (mean *BMI* = 15.7, *SD* = 1.27). As BMI was shown to predict the relative reinforcing value of energy-dense snack food products (Goldfield et al., 2011), it is of specific interest to further investigate possible interaction effects between child-direct labels and nutritional status. Hunger levels affected the liking ratings, but did not affect effort provision. Also, hunger did not moderate or mediate the effect of label on liking or effort provision. These effects should be interpreted with caution, as hunger levels showed little variance in our sample. A previous study, which, in contrast to our study, used food deprivation, showed that hunger level selectively influenced wanting measures (Epstein et al., 2003). Another study found that hunger has little effect on liking ratings. They also observed that when participants rate liking of food products, they often confuse pleasantness of the taste with pleasantness of eating it, and only the latter decreases with eating and is affected by hunger levels (Rogers and Hardman, 2015).

Nonetheless, certain limitations have to be considered. This study included only children aged 8–10, who are possibly not as vulnerable as younger children to marketing cues. However, a recent study showed strong perception biases in response to an external cue, that is food portion sizes, in this age cohort, and marketing effects have been reported in even older children (Cornil et al., 2014; Dixon et al., 2014). Child-directed marketing might be more appealing to overweight children than to their leaner counterparts (Halford et al., 2008; Keller et al., 2012). It is therefore of specific interest to further investigate possible interaction effects of child-directed marketing strategies of healthy products, nutritional status and long-term health outcomes. Only one product was tested, therefore future studies are needed to elucidate the effect of fun labels on a greater variety of healthy food products, especially those that may be more difficult to render attractive to children, such as vegetables. A previous study suggested that the effect of licensed cartoon characters on food choice is smaller for vegetables (Roberto et al., 2010). We also expect a possibly somewhat smaller effect of fun characters on products with a very low energy density. Future research is needed to further analyze the interaction between marketing cues and a product's energy density.

Further, we applied a within-subject design, therefore, children could compare the products. We opted for this design, as it has been previously used for studies on marketing placebo effects (i.e., Allison and Uhl, 1964; McClure et al., 2004; Plassmann et al., 2008). However, this does not correspond to a real-life situation, where one is exposed to a single product at a time and we cannot rule out comparison and demand effects. It is therefore of interest to investigate the effect of package design on taste and effort provision placebo effects in a between-subject design. The effort task always occurred before the liking task. Therefore, the effort task may have influenced the liking ratings. Correlations between placebo effects are significant. However, when correlating the products they liked most with the products children provided most effort for, we did not find a significant correlation (*p* > 0.8). Therefore, the effect of effort provision on the liking task seems to be rather small.

Another limitation of the present study is the design of the fun label, which was created by adding a cartoon character on a health label. The health labels “OptiMix” and “Optimal snack” were intended for parents and are mostly unknown in Germany. It is of course conceivable that the observed effect is due to salience or the combination of fun characters and health information. However, the effects of the health logo alone on taste pleasantness ratings and effort provision are not very strong; we therefore assume that the interaction effects are also rather small. Previous studies claimed that only few children around the age of 8 years (such as in our study) have internalized health concerns (e.g., Cornil et al., 2014). Another study has shown that children (12 years) can differentiate between healthy and unhealthy foods, but although this knowledge is a prerequisite for making healthy choices, it was shown to be insufficient to promote healthier dietary choices (Douglas, 1998). A study in adults showed that a health label can make a food product even less appealing and decrease its rewarding properties (Ng et al., 2011), which we cannot find in our cohort. This may be due to the additional fun character, an absence of interest in or knowledge of the health label, differences in age characteristics, or other factors. However, in light of a previous review (Jenkin et al., 2014), nutrition and health claims may also affect children. This has to be investigated in future studies, as prior research suggests an interaction between health and hedonic attributes in food choice (Wansink and Chandon, 2006; Cornil et al., 2014). In addition to adding a cartoon character to the fun label, we also changed the name from a rather official, and potentially not very common product name for children in Germany (i.e., “Früchtejoghurt mit Cerialien,” English: “Fruit yogurt with cereals”), to “Knabbadus,” a child-directed product name. We intended that both aspects should increase the “fun” component of the product. The observed effects may be therefore due to a combination of cartoon character and child-directed product name or due to removing a rather official name from the plain and health-only labeled product. Future studies should disentangle whether the observed effect is due to a combination of cartoon character and attractive product name, or can also be achieved by manipulating only one of the components.

It is important to note that the handgrip strength measure was cued, in that participants were told that hand grip strength should correspond to how much they wanted the product. This is a quite explicit measure, as children could consciously manipulate the amount of effort they were willing to expend. Future studies are needed to determine if the reinforcing value could be adequately captured with this instruction, for example by using a more implicit setup. Using a more spontaneous measure, such as the effort expended to grasp different kind of foods, may greatly improve our understanding. Although it is not possible to completely rule out the possibility of demand effects, that is, changes in behavior due to cues about what constitutes appropriate behavior (Zizzo, 2010), several steps were taken to prevent this bias. Product samples were randomly ordered, and children did not receive feedback about their selections. Further, demand effects are expected to be much lower in children as their ability to understand persuasive intents is lower than in adults (Roberto et al., 2010). Finally, one would expect that demand effects are less visible in effort provision tasks compared to self-reported data, such as pleasantness ratings (Mogg et al., 2003; Steffens, 2004). However, we obtained similar results for both measures. Future research is also needed to describe the specific mechanisms by which cartoon characters influence children's preferences (Kraak and Story, 2015).

A systematic review concluded that food promotion by the industry has a major impact on children's preferences, food choices and purchase requests (Hastings et al., 2003). Importantly, the nutritional quality of promoted food products appears to correlate with the nutritional quality of product purchases (French et al., 2001). Therefore, strategies and techniques used by the food industry could improve public policy interventions that aim at making healthy food choices more appealing to children in order to mitigate the gap between dietary recommendations and food choice. Target-specific marketing of healthy food products may also be promising for industry-initiated efforts to increase sales of healthy items. Many campaigns promoting healthy eating patterns are based on an educational approach and focus on cognitive abilities, but increased food knowledge does not automatically lead to healthier food choices (Kopelman et al., 2007; Reisch et al., 2013). Future intervention strategies should also focus on child-directed marketing cues that are shown to increase healthy choices. Finally, it is of utmost importance to reduce the gap between food-related research and effective public policy interventions (McCarthy et al., 2011). The availability of a feasible and valid instrument to investigate the impact of marketing cues on children's healthy food choice may improve the implementation and monitoring of evidence-based intervention strategies. Improved understanding of marketing actions and the ability to assess both individual wanting and liking of healthy food products could ultimately foster new research and significantly improve policy interventions.

In sum, our results suggest a pronounced effect of child-directed marketing cues with a fun label on healthy food products on children's taste perception and their willingness to provide effort to obtain a snack food item. Using marketing cues to promote healthy food products to children may prove to be a promising strategy to increase healthy choices in children, however, long-term effects need to be studied in more detail. Further, we present a novel tool in food marketing research, that is, the handgrip dynamometer to measure effort provision. Results suggest that effort provision may capture additional variance in food choice, and may prove to be an interesting tool for future field studies as well as for the evaluation of policy interventions.

### Conflict of interest statement

The authors declare that the research was conducted in the absence of any commercial or financial relationships that could be construed as a potential conflict of interest.
